# Extraction of gymnemic acid from *Gymnema inodorum* (Lour.) Decne. leaves and production of dry powder extract using maltodextrin

**DOI:** 10.1038/s41598-023-38305-4

**Published:** 2023-07-11

**Authors:** Rojarej Nunta, Julaluk Khemacheewakul, Sumeth Sommanee, Chatchadaporn Mahakuntha, Mayuree Chompoo, Yuthana Phimolsiripol, Kittisak Jantanasakulwong, Anbarasu Kumar, Noppol Leksawasdi

**Affiliations:** 1 Cluster of Agro Bio-Circular-Green Industry (Agro BCG) & Bioprocess Research Cluster (BRC), School of Agro-Industry, Faculty of Agro-Industry, Chiang Mai University, Chiang Mai, 50100 Thailand; 2 Division of Food Innovation and Business, Faculty of Agricultural Technology, Lampang Rajabhat University, Lampang, 52100 Thailand; 3 Faculty of Agro-Industry, Chiang Mai University, Chiang Mai, 50100 Thailand; 4 Department of Biotechnology, Periyar Maniammai Institute of Science & Technology, Thanjavur, 613403 India

**Keywords:** Biotechnology, Plant sciences

## Abstract

The aim of the present study was to maximize the extraction of gymnemic acid (GA) from Phak Chiang Da (PCD) leaves, an indigenous medicinal plant used for diabetic treatment in Northern Thailand. The goal was to overcome the low concentration of GA in the leaves, which limits its applications among a larger population and develop a process to produce GA-enriched PCD extract powder. The solvent extraction method was employed to extract GA from PCD leaves. The effect of ethanol concentration and extraction temperature were investigated to determine the optimum extraction conditions. A process was developed to produce GA-enriched PCD extract powder, and its properties were characterized. In addition, color analysis (L*, a*, and b*) was performed to evaluate the overall appearance of the PCD extract powder. Antioxidant activity assay was conducted to assess the ability of the PCD extract powder to neutralize DPPH free radicals. The results showed that the concentration of 50% (*v/v*) ethanol at 70 °C for 2 h resulted in a higher GA concentration of 8307 mg/kg from dried PCD leaves. During the drying process, the use of maltodextrin at a concentration of 0.5% (*w/v*) was found to produce PCD extract powder with the maximum GA concentration. The color analysis revealed that the PCD extract powder had a dark greenish tint mixed with yellow. The antioxidant activity assay showed that 0.1 g of PCD extract powder was able to neutralize 75.8% of DPPH free radicals. The results concluded that PCD extract powder could potentially be used as a source of nutraceuticals or as a functional food ingredient. These findings suggest the potential value of GA-rich PCD extract powder in various applications in the pharmaceutical, nutraceutical, or food industries.

## Introduction

Gymnemic acid (GA) is a complex group of molecules found in the leaves of *Gymnema* sp.^1^ and known for its hypoglycemic effects, making it a popular natural treatment for diabetes^2,3^. Due to the structural similarity with glucose, GA can bind to the sweet taste receptors on the taste buds, effectively competing with sugar molecules for receptor binding. As a result, the sensation of sweetness is hindered, as GA occupies the receptors and prevents their activation by sugar molecules. This effect can help reduce sugar cravings and may help control blood sugar levels in people with diabetes^4^. In addition to its potential anti-diabetic effects, GA has also been studied for its anti-inflammatory, anti-obesity, and anti-cancer properties^3,5^.

In this regard, *Gymnema inodorum*, indigenous to Thailand, one of the local Thai vegetables, found everywhere in the northern region and locally named “Phak Chin Da” or “Phak Chiang Da” (PCD) have been used as a traditional medicine for the management of diabetes^6–8^. While the leaves of the *G. inodorum* plant have been used for centuries in traditional medicine to treat various ailments, using whole leaf extract has some disadvantages that should be considered. For instance, Gymnema extract can contain varying amounts of GA, which can make it difficult to achieve consistent dosing. The extract can contain other compounds that may interact with medications or have other side effects. Moreover, some compounds in whole leaf extracts may hinder the absorption of GA by the body, reducing its bioavailability and effectiveness^9^.

To overcome this, there have been several research articles that have investigated the extraction and quantification of GA from *Gymnema* leaves using various extraction methods and solvents. Few studies found that ultrasound assisted extraction of *G. sylvestre* leaves was efficient and faster technique in extracting GAs and other bioactives^10–14^ when compared to other conventional methods. On the other hand, Mandal et al.^15^, found that microwave-assisted extraction was superior than other extraction methods such as heat flux extraction, maceration and stirring extraction for the recovery of GAs. Although several extraction methods have been described in the literature, solvents-based extraction is the most widely used method where the solvent penetrates the solid matrix solubilizing the solute that diffuses out of the matrix. While some researchers have demonstrated that solvent-based Soxhlet extraction yielded highest GAs from *G. inodorum* leaves^16,17^, others have stated that solvent maceration-based extraction was suitable for the recovery of GAs^18–23^. In general, solvent-assisted extraction methods have several advantages over other methods in terms of higher extraction efficiency, selectivity and reduced energy consumption.

The extraction of GA from *Gymnema* leaves can be achieved using different solvents, including water, methanol, ethanol, and acetone. The choice of solvent may depend on various factors, including the solubility of GA in the solvent, the extraction efficiency, and the safety and environmental impact of the solvent. Generally, polar to medium polar solvents are used to extract phenolic acids, alkaloids, flavonoids and tannins which are polar in nature. In this study, ethanol, an organic polar solvent was employed in the extraction of GA due its polarity^24^. It was also reported that a combination of polar solvents such as ethanol and water at different proportions can be ideal to extract phenolic acids since both the solvents have different polarity values (ethanol: 0.654, water: 1.0) and their acceptability for human consumption^25^. In addition to the choice of the solvent, several parameters such as solvent concentration, extraction temperature, solvent-to-solid ratio and extraction duration may significantly influence the extraction efficiency and concentration of bioactive compounds^26^.

Although several reports have been published on the preparation of extracts from PCD leaves^27–29^, there is scarce information on the preparation of powder formulation utilizing the extracts. The idea of preparing a powder formulation from the extracts of PCD leaves will more likely to gain popularity in the food and nutraceutical industry due to added benefits. Generally, to prepare a powder from the extracts, carriers such as maltodextrin^30,31^, starch^31,32^, and cellulose derivatives^33,34^ are used during drying process. The selection of a carrier may depend on several factors such as the physical and chemical properties of the extract, solubility and stability of the active compounds, the compatibility of the carrier with the active compounds in the plant extract and the intended application^35–37^. Maltodextrin is preferred over other carriers for their excellent flowability and dispersibility. It can reduce moisture reabsorption and reduce powder particle agglutination, make the extract powder retain its shape for a longer time and extend the shelf life^30^. Moreover, the addition of maltodextrin can have the advantages over other drying agents for its cost-efficient, highly soluble and poorly hygroscopic characteristics^30,38,39^, thus chosen in the present study.

The aim of the present study is to optimize the extraction conditions for higher recovery of GA from *G. inodorum* leaves and to develop a process for the production of PCD dried extract powder.

## Materials and methods

### Plant material and chemicals

The medium-matured leaves of *G. inodorum* (Lour.) Decne. were procured from Wor Kaew Subdistrict, Hang Chat District, Lampang Province, which is a well-known area for commercial farms growing *G. inodorum* in Northern Thailand. The leaves were collected between January and February 2022 using species No. 6 from certified Agricultural Land Plot No. DOA-52120700-9058-0006 with permission from the owner of this Land Plot under supervision of Department of Agriculture for specimen collection. The use of this economic plant, which was not endangered or protected species, complied with international and national guidelines of Good Agricultural Practices (GAP) for Food Crops/Thai Agricultural Standard (TAS) 9001(G)-2021. All records of specimens and Agricultural Land Plot usage were kept at Plant Standard and Certification Division, Department of Agriculture, Ministry of Agriculture and Cooperatives, Bangkok, Thailand. The ethanol used for extraction in this study was food grade with 99.8% (*v/v*) purity (Liquor Distillery Organization, CAS No. 64-17-5, Chachoengsao, Thailand). All other chemicals utilized were either Analytical or HPLC grades.

### Preparation of dried PCD leaves powder

PCD leaves powder was prepared as described by Ounjaijean et al.^40^ with slight modifications. Briefly, PCD leaves were washed thoroughly, drained, and dried in a hot air dryer (Owner Foods Machinery Co., Ltd., Model SO 6A, Bangkok, Thailand) until a constant weight was obtained. Dried leaves were ground with a grinder mill (Huang Cheng, Model 4500A, China) and sieved through a sieve shaker (Endecotts, Model Octagon 200, London, United Kingdom) using a 100-mesh sieve size to yield the particle size not more than 150 µm.

### Preparation of PCD extract powder

**Figure 1:**
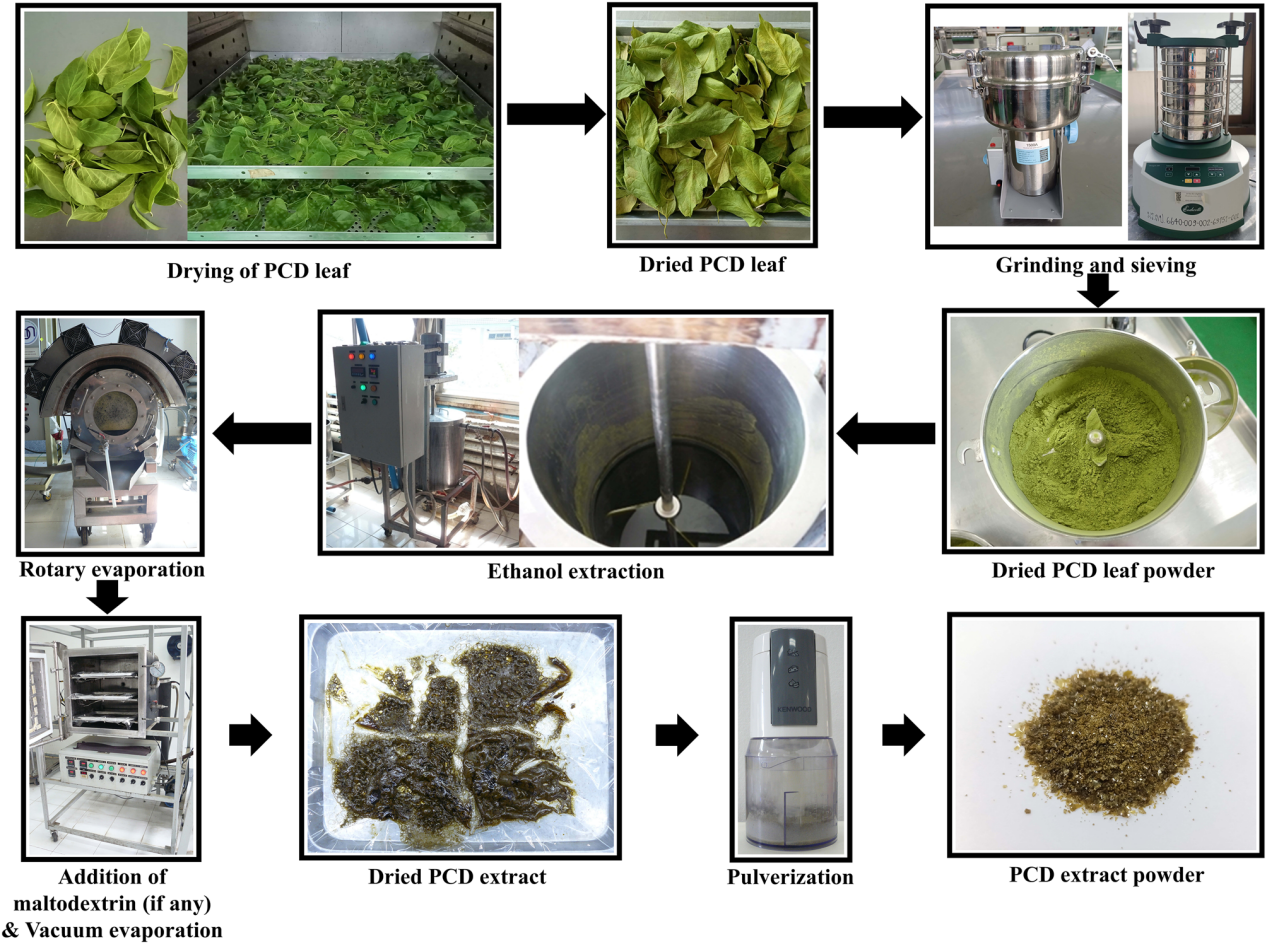
The production process of PCD extract powder from PCD leaves.

PCD extract powder preparation was adapted from Jiménez-Moreno et al.^41^, Fig. 1 shows the step-by-step process of PCD extract powder production. Briefly, the PCD leaves were dried at optimized drying temperature (“Effect of PCD leaves drying temperature” section) prior to attrition process with industrial high-speed grinder (Huang Cheng, Model 1500A, China) with screen size of 100 mesh (Endecotts, Model Octagon 200, London, United Kingdom) to attain PCD leaves powder. Further, 1 kg leaves powder was suspended in 20 L ethanol at the optimized concentration (“Effect of ethanol concentration during GA extraction from dried PCD leaves powder” section) in a 50 L double jacketed continuous stirring bioreactor (FEBIX International, Model ST-50L, Chiang Mai, Thailand) and stirred at a speed of 50 rpm for 2 h. The extraction was carried out at optimized temperature (“Effect of temperature during GA extraction from dried PCD leaves powder” section). The resultant slurry was filtered with double layer muslin cloth and the ethanol fraction was concentrated using a rotary evaporator (FEBIX International, Model REvap001, Chiang Mai, Thailand) at 50 °C and 10 rpm. When 50% (*v/v*) of the ethanol volume was evaporated, maltodextrin was added at the optimized concentration (“Effect of maltodextrin concentration during production of PCD extract powder” section) to the concentrated extract. The extract was then evaporated to dryness at 65 °C using a laboratory-scale vacuum dryer equipped with a diaphragm vacuum pump (Vacuubrand, Model MZ 2C NT + AK + EK, Wertheim, Germany) for creating the necessary vacuum (76 ± 5 cm Hg) inside the drying chamber. The dried material was pulverized into powder using an electric food chopper (Kenwood, Model CH550, Hampshire, UK).

### Experimental design

In this study, firstly, a single factor experiment was used to optimize the extraction of GA from PCD leaves by studying the effects of three factors, namely drying temperature, ethanol concentration and extraction temperature^41^. In this experiment, one of the parameters is changed at a time while the other parameters are kept constant. Secondly, the PCD extract obtained using optimized conditions was processed further into PCD extract powder and the maltodextrin concentration was optimized during the preparation. Finally, the PCD extract powder prepared with optimized concentration of maltodextrin was evaluated for its GA, chlorophylls content, water activity (a_w_), moisture content, color (L*, a*, b*) and antioxidant capacity by 2,2-diphenyl-1-picrylhydrazyl (DPPH) method. The details are described in the analytical section.

### Effect of PCD leaves drying temperature

Three different temperature levels, 40, 50 and 60 °C were applied during the drying process of PCD leaves while the concentration of ethanol and extraction temperature were kept constant at 50% (*v/v*) and 70 °C, respectively during extraction of GA from dried leaves. The levels of drying temperature used in this study were selected based on the literature where the authors have reported that drying at above 70 °C was detrimental to the bioactive properties and temperature up to 60 °C retained most of the bioactive compounds^42,43^. The best drying temperature was chosen based on the color L* a* b* and GA content of dried leaves. For GA extraction, PCD leaves powder obtained from each drying temperature level was extracted with 50% (*v/v*) ethanol at a 1:20 (*w/v*) ratio in a water bath (Memmert, Model WNB 7-45, Germany) at 70 °C for 2 h under static conditions. The concentration of ethanol, extraction temperature and duration were selected from our previous preliminary research where solvent concentration above 50%, extraction temperature above 70 °C and duration beyond 2 h resulted in decreased GA content of *G. inodorum* cultivated in different region (unpublished data). The slurry after extraction was filtered through a double layered muslin cloth to separate the residue and extract. The extract was filtered using an Agilent PTFE syringe filter (0.45 µm) and aliquot was transferred to an HPLC vial. The content of GA in the extracts was quantified with HPLC as described in the analytical section.

### Effect of ethanol concentration during GA extraction from dried PCD leaves powder

Ethanol was chosen for the extraction of GA as it considered to be a food grade solvent^44,45^ and moreover it was found to be the appropriate solvent by virtue of safety and efficiency^46^. The effect of ethanol concentration on GA extraction was studied using the PCD leaves dried at best drying temperature chosen from the previous step and pulverized. Six levels of ethanol concentration, namely 0, 20, 30, 40, 50 and 60% (*v/v*) prepared in distilled water were used for the extraction of GA from PCD leaves powder while the extraction temperature was maintained at 70 °C. The slurry after extraction was processed as stated above and quantified for GA content. The minimum ethanol concentration that resulted in the optimized GA content and extraction yield was utilized for further experiments.

### Effect of temperature during GA extraction from dried PCD leaves powder

The PCD leaves dried at best drying temperature determined in the first step and powdered was used to select the suitable extraction temperature. PCD leaves powder was extracted by holding at different extraction temperature namely, 40, 50, 60, 70 and 80 °C for 2 h under stationary condition while the concentration of ethanol was kept constant at the best concentration chosen from the second step. The extracts were then filtered through muslin cloth and GA content in the filtrate was quantitated as mentioned earlier. The best extraction temperature was chosen according to the optimized GA content and extraction yield.

### Effect of maltodextrin concentration during production of PCD extract powder

The PCD extract powder was produced by drying the PCD leaves at optimum drying temperature followed by powdering and extracting with optimum ethanol concentration at optimum extraction temperature. Methods described by Lachowicz et al.^47^ and Widyaningsih et al.^48^ were adapted for the production of PCD extract powder using maltodextrin with slight modifications. Different concentrations of maltodextrin ranging from 0 to 5.0% (*w/v*) were used for the study and added to the concentrated slurry which was concentrated by two times after rotary evaporation stage. The upper concentration of maltodextrin was restricted to 5% considering its high glycemic index. For the extraction of GA, PCD extract powder prepared with or without maltodextrin was suspended in 50% (*v/v*) ethanol, centrifuged twice at 2822×*g* (Nuve, Model NF 200, Ankara, Turkey) for 5 min followed by 6742×*g* (Eppendorf, Model Mini spin, Hamburg, Germany) for 1 min and filtered through Agilent PTFE syringe filter (0.45 µm) before HPLC analysis. The suitable maltodextrin concentration was chosen based on the higher GA content and extraction yield.

### Analytical methods

#### Color analysis

Color parameters of samples were measured using a color meter (Minolta, Model CR-300 chroma meter, Osaka, Japan) and reported as L*, a*, b* values. L* indicates lightness (100) or darkness (0), a* means red (+) or green (−) and b* represents chromaticity yellow (+) or blue (−)^49,50^.

#### Determination of moisture content

The moisture content on mass basis was determined as per the 17th edition of Association of Official Analytical Chemists (AOAC)^51^. Briefly, a known amount of sample was weighed and dried in a hot air oven (Daihan Lab Co., Ltd., Gangwon, Korea) at 105 °C for 4–5 h until constant weight to determine the moisture content and calculated according to the Eq. (1) ^52^.

1 $$\% {\text{ Moisture content }} = \, \left[ {\left( {{\text{Initial weight }} - {\text{ Final weight}}} \right)/{\text{initial weight}}} \right] \, \times { 1}00.$$

#### Determination of water activity (a_w_)

The a_w_ of the samples was performed using a water activity meter (AQUA Lab, Model CX3TX, Washington, USA). The a_w_ was determined from the triplicate samples (2 g) held at 25 ± 0.1 °C until equilibrium was reached^52^.

#### Quantification of chlorophylls content

For the extraction of chlorophylls, 0.5 g of PCD extract powder was mixed with 10 mL of ice-cold acetone and shaked in an orbital shaker (LabTech, Model LSI 3016R, Korea) at 250 rpm for 1 h at 30 °C. Later, 5 mL of ice-cold ethanol was added to it and shaked again at the similar condition. The samples were filtered and the extraction process were repeated until the sample residue become colorless. The extracted samples were pooled together and concentrated under vacuum at 65 °C^53^. The residue was re-dissolved in HPLC-grade absolute methanol (1 mL) before analyzed by HPLC. Chlorophylls content were determined using a reversed-phase HPLC system equipped with a degasser, autosampler and diode array detector (Agilent Technologies, Model 1260 Infinity series, Santa Clara, California, USA). HPLC analysis of chlorophylls was performed according to the method described by Varzakas and Kiokias^54^ with some modifications. The column used for chlorophylls detection was an Agilent Poroshell 120 SB-C18 with a specification of 2.1 × 100 mm, 2.7 µm which was maintained at 30 °C. The isocratic mobile phase consisted of ethyl acetate: methanol (20:80, *v/v*) at the flow rate of 0.60 mL/min with a run time of 15 min and the injection volume was 5 µL. The spectra were recorded and the chromatograms were obtained at 430 nm for chlorophyll a and 474 nm for chlorophyll b. The identification of chlorophylls was based on the respective standards and their retention times. The identified compounds were quantitated from the peak area using respective calibration curve prepared in the range from 0 to 200 ppm (1 ppm = 1 mg/L). The chlorophylls content calculated in ppm was converted into mg/L of extract and expressed as mg/kg on dry weight basis by considering the initial dried weight of powder used for the extraction.

#### Quantification of GA content

GA content was determined using a reversed-phase HPLC system equipped with a degasser, autosampler and diode array detector (Agilent Technologies, Model 1260 Infinity series, Santa Clara, California, USA). HPLC analysis of GA was performed according to the method described by Salunkhe and Magdum^55^ with some modifications. The column used for GA detection was an Agilent Zorbax Eclipse Plus C18 with a specification of 4.6 × 100 mm, 3.5 µm which was maintained at 40 °C. The gradient mobile phase consisted of solvent A as 1% (*v/v*) phosphoric acid and solvent B as absolute methanol (HPLC grade). The flow rate was fixed at 0.65 mL/min and the injection volume was 5 µL. The gradient elution program was started with 90% (*v/v*) A and 10% (*v/v*) B (0 min). At 4 min, the gradient reached was 30% A (*v/v*) and 70% B (*v/v*) and maintained for 12 min. The gradient then finally reached 90:10% (*v/v*) (A: B) at 16 min and maintained for 4 min with a total run time of 20 min. The detection was carried out at 210 nm^55^. The identification of GA was based on the standard and its retention time. The identified compounds were quantitated from the peak area using respective calibration curve prepared in the range from 0 to 100 ppm. The GA content calculated in ppm was converted into mg/L of extract and expressed as mg/kg on dry weight basis by considering the initial dried weight of powder used for the extraction. Mass yield percentage of extracted GA (%Y_GA_) was calculated as shown in Eq. (2).

2 $$\% {\text{Y}}_{{{\text{GA}}}} = \, \left[ {{\text{GA content }}\left( {{\text{mg}}/{\text{kg}}} \right) \, / \, \left( {{1}000{\text{ g }} \times { 1}000{\text{ g}}} \right) \, \times { 1}00\% } \right]$$

Improved purity percentage of GA content in PCD extract powder relative to dried PCD leaves powder (% P_Ext/Leaves_) was calculated as shown in Eq. (3).

3 $$\% {\text{P}}_{{{\text{Ext}}/{\text{Leaves}}}} = \, \left[ {\left( {{\text{GA content of PCD extract powder }}{-}{\text{ GA content of PCD leaves powder}}} \right)/{\text{GA content of PCD leaves powder}}} \right] \, \times { 1}00$$

#### DPPH scavenging activity

DPPH free radical scavenging capacity of PCD leaves extract powder was analyzed as previously described by Meza et al.^56^. Briefly, a 0.1 mM ethanolic stock solution of DPPH was prepared and diluted ten times to obtain the working solution. A 300 µL of sample solution containing PCD extract powder suspended in absolute ethanol at 10 mg/mL concentration was mixed with DPPH working solution (1600 µL). The mixture was incubated in the absence of light for 1 h and absorbance was measured at 515 nm using a UV–Vis spectrophotometer (Agilent Technologies, Model Cary 60 UV–Vis, Santa Clara, California, USA). Ascorbic acid and gallic acid dissolved in absolute ethanol at 10 mg/mL were used as positive controls for comparing the antioxidant activity. The percentage of radical scavenging activity (%RSA) was calculated according to the Eq. (4) ^57^.

4 $$\% {\text{ RSA }} = \, \left[ {\left( {{\text{A}}_{0} - {\text{A}}_{{{6}0}} } \right)/{\text{ A}}_{0} } \right] \, \times { 1}00$$

where A_0_ is the absorbance of DPPH radical solution at 515 nm without extract or standard at 0 min; A_60_ is the absorbance of DPPH radical solution at 515 nm in the presence of extract or standard at 60 min.

### Statistical analysis

Three replications were carried out in each experiment and the results were expressed as mean ± standard error. Statistical analyses were carried out as described in our earlier studies^58–60^ with Statistical Packages for the Social Sciences (SPSS, version 17.0) applying one-way analysis of variance (ANOVA). Statistical differences between means were measured using Duncan's multiple range test (DMRT) and considered significant at p ≤ 0.05.

## Results

### Effect of PCD leaves drying temperature

**Figure 2:**
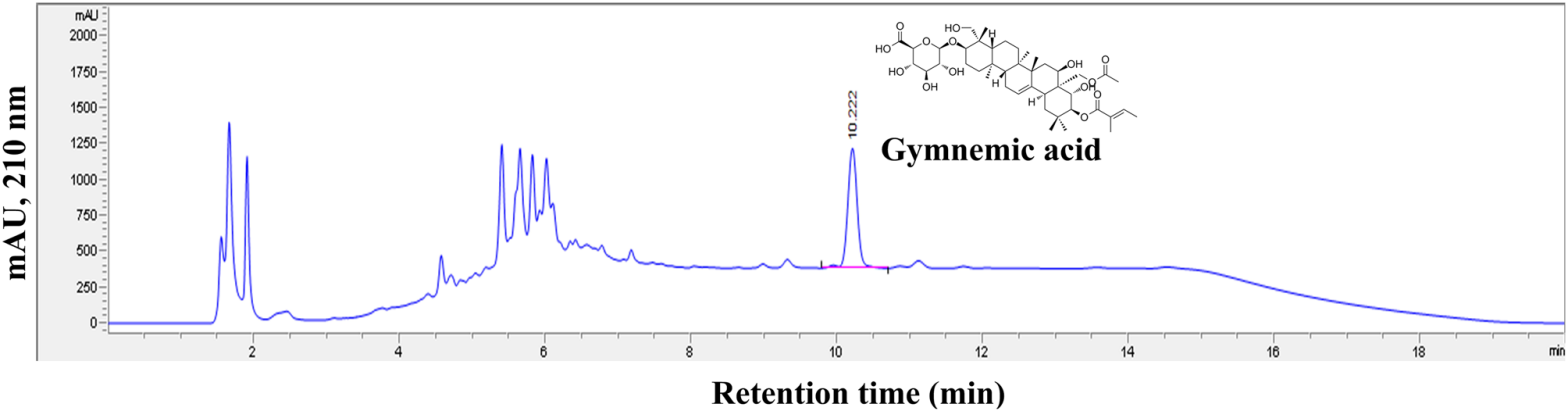
HPLC chromatogram of GA present in PCD leaves.

The GA content presented in PCD leaves when dried at 40, 50 and 60 °C was analyzed through HPLC (Fig. 2) and shown in Table 1. The dried leaves at 40 and 50 °C had a moisture content of no more than 5.0 ± 0.5% (*w/w*). The GA content of PCD leaves was significantly (p ≤ 0.05) influenced by drying temperature beyond 40–50 °C range and tended to decrease with increased temperature. For instance, when the drying temperature increased to 60 °C, the GA content significantly (p ≤ 0.05) decreased by 5% (*w/w*) when compared to drying at 50 °C. However, the GA content (8307 ± 39 mg/kg) presented in PCD leaves when dried at 50 °C was not significant (p > 0.05) when compared to drying at 40 °C.

**Table 1:** Appearance and GA content of dried PCD leaves at different temperatures.

Drying temperature (°C)	Duration (h)	GA content (mg/kg dried leaves)	Appearance
40	24	**8224**^**A**^** ± 40**	
50	12	**8307**^**A**^** ± 39**	
60	8	7882^B^ ± 21	

Numbers with the same alphabet indicated no significant difference (p > 0.05) for comparison of the same column.

*PCD* Phak Chiang Da, *GA* gymnemic acid.

Bold data indicated the best average value(s) within each column.

**Figure 3:**
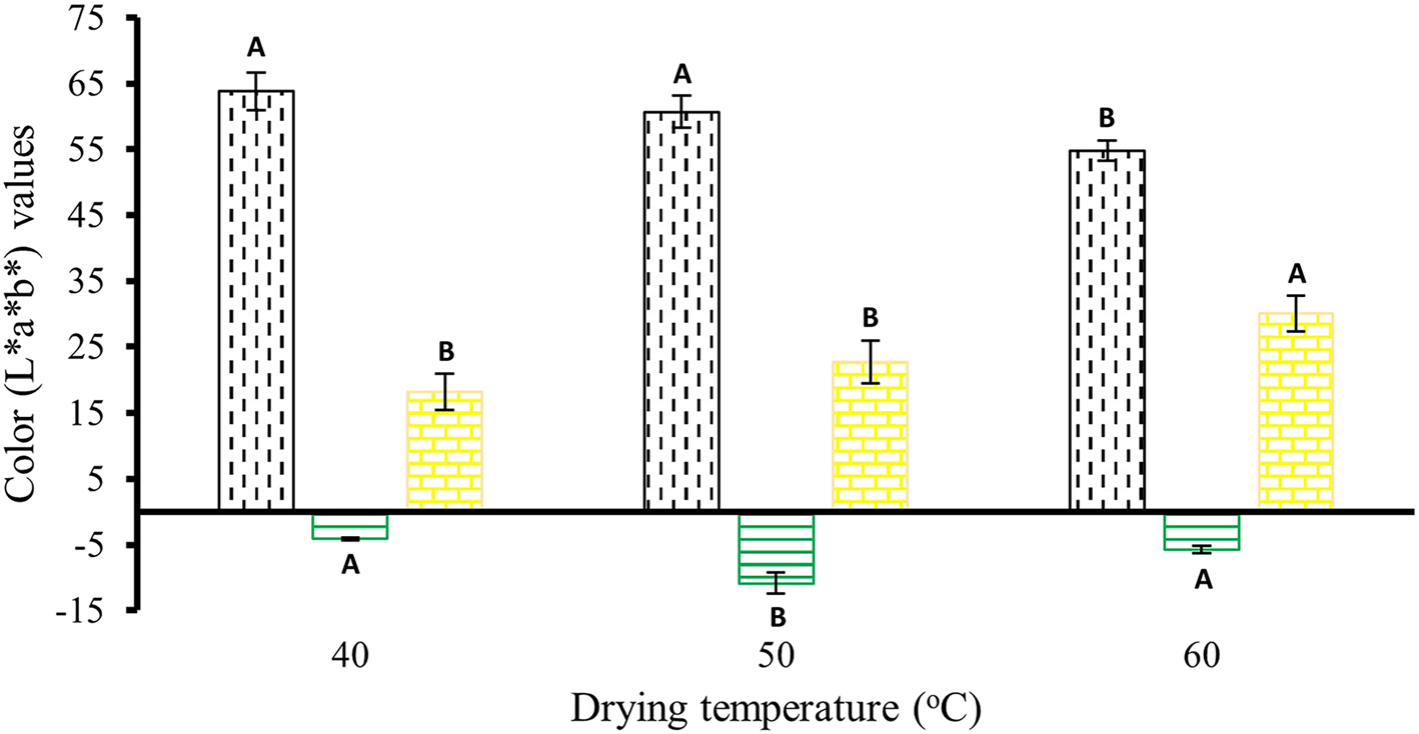
Effect of drying temperature on color parameters of PCD leaves. The symbol “” represents a spectrum of lightness and darkness, ranging from 0 (representing black) to 100 (representing white). The symbol “” signifies the spectrum of green (− 60) to red (+ 60), while “” represents the spectrum of blue (− 60) to yellow (+ 60). Error bars indicate the standard errors which deviated from the mean (n = 3). The average values with different alphabets indicated a significant difference (p ≤ 0.05) among the same type of color value between drying temperatures.

Color is an important parameter in which L* indicates a light–dark spectrum with a range from 0 (black) to 100 (white), a* denotes the green (-60) to red (+ 60) spectrum, and b* means the blue (− 60) to yellow (+ 60) spectrum dimensions^49^. The different temperatures in the drying method had a significant effect (p ≤ 0.05) on the lightness (L*), redness (a*), and yellowness (b*) of PCD leaves. As illustrated in Fig. 3, L* values decreased significantly (p ≤ 0.05) from 63.8 to 54.8 when the drying temperature increased from 40 to 60 °C. The lesser the L* value, the darker the leaves became. It was also observed that the color of the leaves gradually shifted in green and yellow directions. This was evident from a decrease in greenness (a*) and an increase in yellowness (b*). However, the leaves dried at 40 °C possessed less green color when compared to 50 or 60 °C. In terms of redness (a*), samples dried at 60 °C showed an increase in positive a* values. Similarly, b* value increased upon increasing temperature. As the drying temperature of 60 °C resulted in the darker color of the leaves when compared to using a lower temperature, using the temperature between 40 and 50 °C is thus suggested for drying. However, considering the energy consumption rate, drying at 40 °C consumes more energy in terms of duration as it requires 24 h for complete drying. On the other hand, drying at 50 °C is considered energy efficient as the drying process is completed within 12 h. The leaves were dried at 50 °C for 12 h with a mass yield of 11.7 ± 1.1% (*w/w*) on a fresh weight basis further ground to powder, and sieved through a siever to yield powder particles of not more than 150 µm in size.

### Effect of ethanol concentration during GA extraction from dried PCD leaves powder

**Figure 4:**
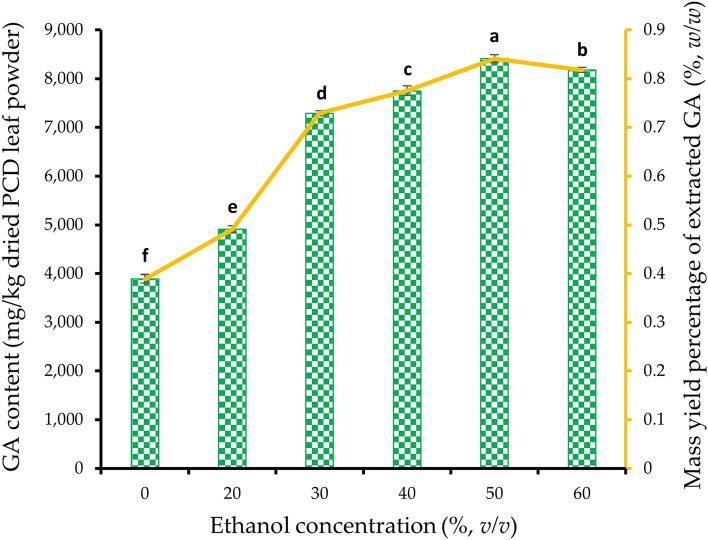
Effect of ethanol concentration on GA content () and mass yield percentage () of extracted GA of dried PCD leaves powder. A constant extraction temperature of 70 °C for 2 h was maintained while varying different concentrations of ethanol. Error bars indicate the standard error from the mean (n = 3). Means with different lowercase alphabets indicated a significant difference (p ≤ 0.05) between ethanol concentrations. *PCD* Phak Chiang Da, *GA* gymnemic acid.

The dried PCD powder obtained from the previous step was extracted with different concentrations of ethanol and the results are presented in Fig. 4. From the results, an increase in GA content was observed with the elevated concentration of ethanol used for the extraction. Distilled water as a solvent (0% ethanol) could extract the least amount of GA with only 3894 ± 87 mg/kg of dried PCD leaves powder. Extraction of PCD leaves powder with 20 to 50% (*v/v*) ethanol resulted in 4912 ± 71 to 8414 ± 82 mg/kg. The Y_GA_ at 50% (*v/v*) ethanol was 2.2-fold higher when compared to GA extracted with distilled water. The %Y_GA_ was found to be 0.84% on dried weight basis of PCD leaves powder. From the results, it was observed that when the concentration of ethanol was increased to 60% (*v/v*), a significant (p ≤ 0.05) drop in GA content was noticed when compared to 50% (*v/v*). From the results, it is suggested that a concentration of 50% (*v/v*) ethanol was the optimum level for extracting 8414 ± 82 mg/kg GA from dried PCD leaves.

### Effect of temperature during GA extraction from dried PCD leaves powder

**Figure 5:**
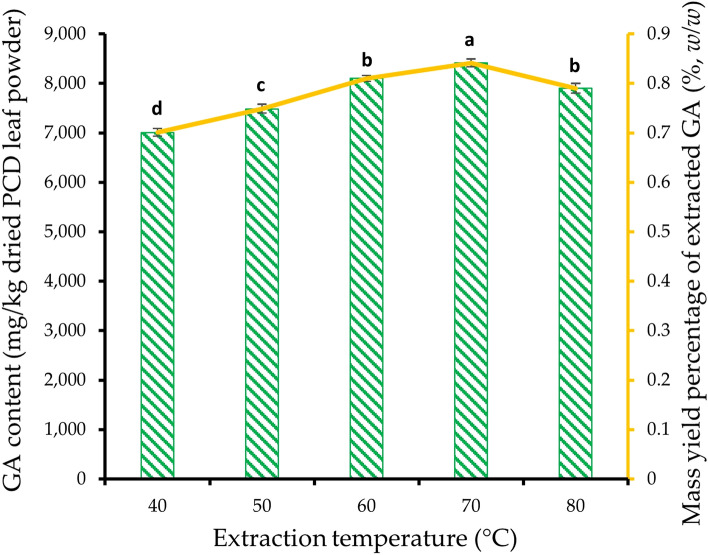
Effect of extraction temperature on GA content () and mass yield percentage () of extracted GA of dried PCD leaves powder. A constant ethanol concentration of 50% (*v/v*) was maintained while varying different extraction temperatures. Error bars indicate the standard error from the mean (n = 3). Means with different lowercase alphabets indicate a significant difference (p ≤ 0.05) between extraction temperatures. *PCD* Phak Chiang Da, *GA* gymnemic acid.

In this experiment, PCD leaves powder was extracted with 50% ethanol as optimized from the previous experiment and extraction temperature was studied ranging from 40 to 80 °C maintained for 2 h. The results of the GA content in the extracts are depicted in Fig. 5. The study found that the concentration of GA tended to increase significantly (p ≤ 0.05) from 7006 ± 78 to 8416 ± 80 mg/kg dried weight with increasing extraction temperature from 40 to 70 °C respectively. The %Y_GA_ increased from 0.7% at 40 °C to 0.84% at 70 °C on dried weight basis of PCD leaves powder. However, when the extraction temperature was set at 80 °C, the GA significantly (p ≤ 0.05) mitigated by 6.14% when compared to that of 70 °C. From the results, it is suggested that the extraction of leaves powder with 50% (*v/v*) ethanol at 70 °C for 2 h is appropriate to yield a higher GA content of 8414 ± 82 mg/kg from dried PCD leaves.

### Effect of maltodextrin content during production of PCD extract powder

#### GA content of PCD extract powder prepared with maltodextrin

**Figure 6:**
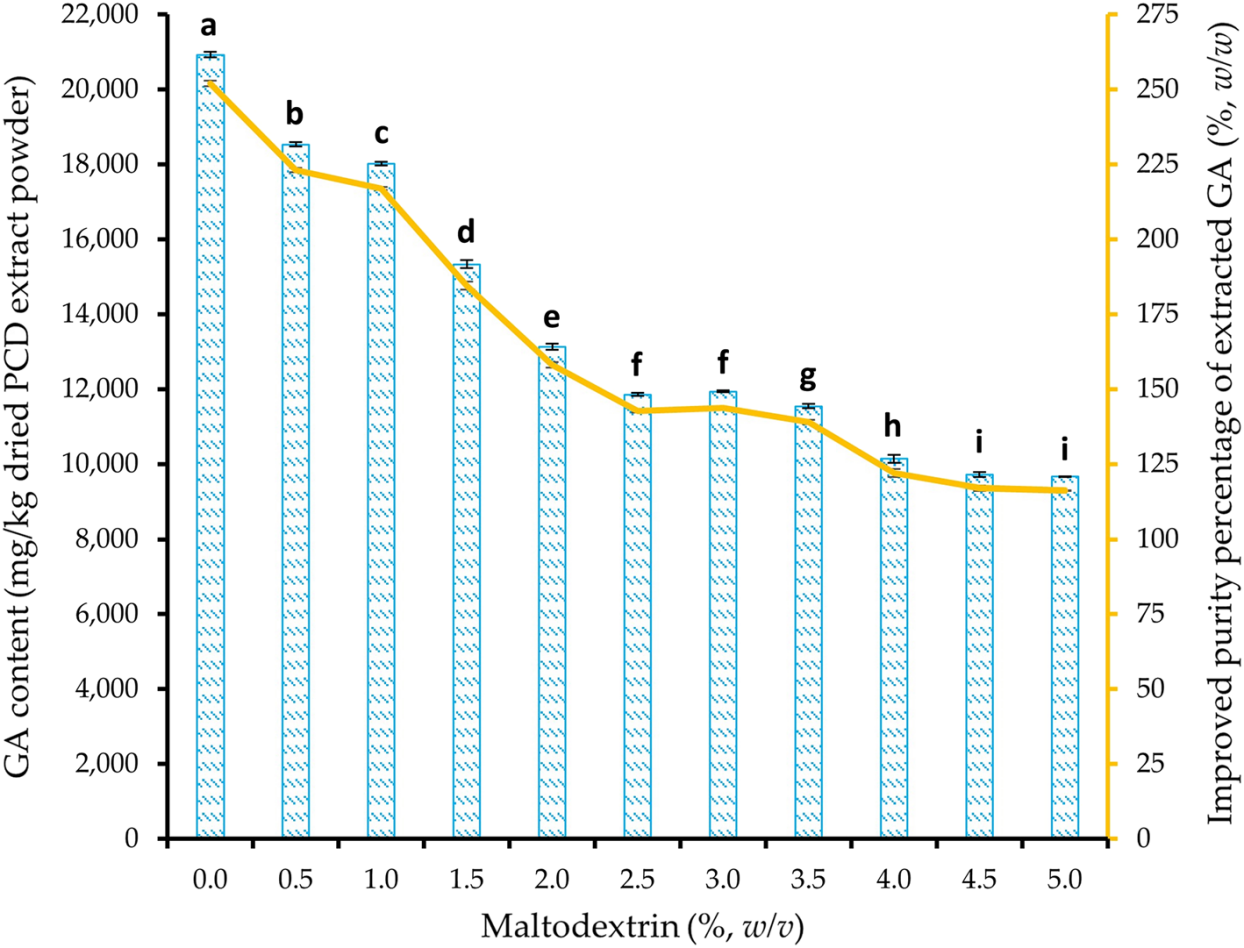
The actual GA content () and improved purity percentage () of GA in PCD extract powder prepared with different maltodextrin concentrations relative to dried PCD leaves powder. Error bars indicate the standard errors from the means (n = 3). The averages with different lowercase alphabets indicate a significant difference (p ≤ 0.05) between maltodextrin concentrations. *PCD* Phak Chiang Da, *GA* gymnemic acid.

Overall, there was an increase in purity percentage of GA in PCD extract powder relative to that of PCD leaves powder. A 252% (*w/w*) of %P_Ext/Leaves_ was seen in purity percentage on mass basis when PCD extract powder prepared without maltodextrin. However, the value gradually decreased from 223% (*w/w*) at the addition of 0.5% (*w/v*) maltodextrin to 116% (*w/w*) with 5% (*w/v*) maltodextrin. The results showed that the amount of GA found in PCD extract powder was inversely related to the amount of maltodextrin used for the preparation. The increased amount of maltodextrin resulted in decreased GA content as shown in Fig. 6. The extract powder prepared without maltodextrin contained a GA content of 20,900 ± 70 mg/kg. From Fig. 6, it was observed that the concentration of maltodextrin added between 0.5 and 5% (*w/v*) had a significant (p ≤ 0.05) effect on the mitigation of the GA content by 53.8% (*w/w*) (9660 mg/kg at 5.0%/20,900 mg/kg at 0% × 100 − 100%). when compared to the powder prepared without maltodextrin. Although the extract powder prepared with 0.5% (*w/v*) maltodextrin affected the GA content, the physical stability of the powder prepared with 0.5% (*w/v*) maltodextrin was retained after 30 days of storage while the powder prepared without maltodextrin caused solid coagulation. Thus, low concentration, 0.5% (*w/v*) maltodextrin could be chosen as an anti-caking agent in the drying process to retain the powder quality during storage.

#### Characterization of PCD extract powder prepared with maltodextrin

The comparison of color parameters, moisture content, a_w_, GA and chlorophylls content between current study and another research^61^ has been depicted in Table 2. From the color analysis, PCD extract powder obtained in the current study had a brightness (L*) value of 47.5 ± 0.21, which is lower when compared to leaves dried at 50 °C indicating that the final product had a darker appearance than starting material. This might be due to the addition of maltodextrin that contributed to the darker appearance when combined with PCD leaves extract at a high temperature (65 °C). Similarly, the mitigation in negative value of a* (− 1.49 ± 0.01) and a positive sign of the b* (14.6 ± 0.3) are indicative of the destruction in green and yellow color of the sample respectively when compared to starting material. Thus, the overall appearance of the PCD extract powder was a dark greenish tint mixed with yellow. Among the chlorophylls, chlorophyll a is more sensitive to pheophytization than chlorophyll b. In the present study, the PCD extract powder contained 356 ± 20 and 270 ± 14 mg/kg of chlorophyll a and b respectively. The GA concentration present in the PCD extract powder prepared with maltodextrin was found to be 18,500 ± 60 mg/kg. The moisture content and a_w_ of the powder sample were found to be 4.76% ± 0.10 (*w/w*) and 0.246 ± 0.001, respectively (Table 2). In general, powders with a moisture content of less than 10% (*w/w*) can be considered microbiologically safe. For instance, in the present study, the PCD powder with 4.76% ± 0.10 moisture had an a_w_ of only 0.246 ± 0.001. This level of a_w_ and moisture content are sufficient enough to prevent the growth of various microorganisms and are ideal for dry or powdered products.

**Table 2:** Color parameters, moisture content, a_w_, GA and chlorophylls content of PCD extract powder.

Parameter	Current study	Binnisha et al.^74^	Jeytawan et al.^76^
L* (no unit)	47.5 ± 0.21	71.18	–
a* (no unit)	− 1.49 ± 0.01	− 6.59	–
b* (no unit)	14.6 ± 0.30	17.72	–
Moisture content (%, *w/w*)	4.76 ± 0.10	7.00	–
Water activity (a_w_) (no unit)	0.246 ± 0.001	N/A	–
GA (mg/kg)	18,500 ± 60	290	102
Chlorophyll a (mg/kg)	356 ± 20	1150	–
Chlorophyll b (mg/kg)	270 ± 14	562	–

*PCD* Phak Chiang Da, *GA* gymnemic acid, *N/A* not available.

Table 3 compares the antioxidant potential of PCD extract powder prepared with 0.5% (*w/v*) maltodextrin with that of Gymnema extracts by others research^23,62–64^. From the results of present study, it was found that PCD extract powder was able to neutralize 75.8% ± 2.6 of DPPH free radicals at 1.58 mg/mL concentration. Results also showed that the standards (gallic acid and ascorbic acid) had stronger scavenging activities against the radical than the PCD extract powder at the same concentration tested.

**Table 3:** Antioxidant capacity of PCD extract powder prepared with 0.5% (*w/v*) maltodextrin by DPPH radical scavenging activity.

Solvent and conditions	% RSA	References
Ethanol (2 h, 70 °C)	75.8 ± 2.6	Current study
Ascorbic acid	93.7 ± 0.4	Current study
Gallic acid	96.8 ± 0.7	Current study
Ethanol (cold maceration method for 72 h at room temperature)	73.4	Subramanian et al.^23^
Methanol (periodic shaking for 5 days, at room temperature)	54.4	Fazal et al.^82^
Methanol (periodic shaking for 1 week at room temperature)	83.0	Ghous et al.^83^
Boiling water (15 min, 80 °C)	62.6	Jeytawan et al.^76^

*PCD* Phak Chiang Da, *RSA* radical scavenging activity.

## Discussion

The study highlights the importance of optimizing the drying temperature for PCD leaves to preserve the GA content and associated antioxidant activity. The results, as shown in Table 1, indicated that the drying temperature had a significant impact on the GA content in the leaves. The dried leaves at 40 and 50 °C with less than 5.0% moisture content suggests that these temperatures were suitable for drying the leaves without retaining excessive moisture, which is crucial for preventing microbial growth and ensuring the stability of the plant material. Drying temperatures within the range of 40–50 °C were found to be favorable for maintaining a higher GA concentration, while temperatures above 60 °C were detrimental to both GA content and overall antioxidant levels. The decrease in GA content observed at 60 °C could be attributed to the oxidation of compounds at relatively high temperatures in the presence of air^65^. When exposed to elevated temperatures, certain heat-labile phytochemicals, including GA, can undergo degradation or chemical changes, resulting in reduced concentrations. This finding aligns with previous reports by Peries et al.^66^ and Sagrin and Chong^67^, which indicated that high drying temperatures can lead to the destruction of heat-labile phytochemicals in plant materials. Furthermore, the study suggested that drying temperatures above 60 °C could significantly mitigate the antioxidant content of the leaves^67,68^. Antioxidants play a crucial role in neutralizing harmful free radicals and preventing oxidative damage. However, excessive heat during the drying process may cause the degradation or denaturation of these antioxidants, leading to a decline in their concentration and thus reducing the overall antioxidant activity of the plant material.

The study also examined the effect of drying temperature on the color characteristics of PCD leaves. Color analysis was conducted using the L*, a*, and b* parameters, which represent the lightness, redness, and yellowness dimensions, respectively. The results presented in Fig. 3 indicated that the drying temperature had a significant effect on the lightness (L*), redness (a*), and yellowness (b*) of PCD leaves. As the drying temperature increased from 40 to 60 °C, the L* values decreased significantly, indicating darker leaves. This darkening of the leaves could be attributed to the degradation of chlorophylls during the drying process^69^. Chlorophylls, responsible for the green color in leaves, undergo degradation when exposed to heat, leading to a decrease in greenness. Furthermore, the color of the leaves gradually shifted in the green and yellow directions. Leaves dried at 40 °C exhibited less green color compared to those dried at 50 or 60 °C, indicating that the degradation of chlorophylls depends not only on the drying temperature but also on the drying duration. The longer drying duration of 24 h at 40 °C, compared to 12 and 8 h at 50 and 60 °C, respectively, likely contributed to the reduction in greenness. This is consistent with the results of Rudra et al.^70^ who reported that drying at higher temperatures and long drying durations are the two important factors resulting in reduced lightness of samples during dehydration process. The increase in positive a* value in samples dried at 60 °C could be attributed to the high temperature exposure, which transforms chlorophylls into pheophytin and pyropheophytin^71^. Similar observations have been reported in studies on other plant materials, such as pandan leaves, where drying at high temperatures led to increased redness. The increase in yellowness (b*) values with increasing temperature could be explained by the degradation of chlorophylls into derivatives caused by the high temperature. This degradation contributes to the brownish color of PCD leaves dried at 60 °C^72^. Overall, the drying temperature and duration play crucial roles in the appearance and color of the leaves during the drying process. The study suggests that a drying temperature between 40 and 50 °C is recommended to preserve the desired color characteristics of the leaves. However, it is worth considering the energy consumption aspect. Drying at 40 °C requires a longer duration of 24 h, while drying at 50 °C completes the process within 12 h, making it more energy-efficient.

When different concentrations of ethanol used for the extraction of GA from PCD powder, the results (Fig. 4) indicate that the GA content increased with higher concentrations of ethanol used for extraction. Comparing the results of the present study with previous research, it is noted that Ahamad et al.^13^ reported a higher %YGA of 12.2% from *G. sylvestre* leaves. The difference in yield could be attributed to the extraction methods used in the respective studies. Ahamad et al.^13^ employed ultrasonic sound in coupled with Soxhlet extraction, while the present study utilized ethanol extraction without ultrasonic sound. Additionally, different species of Gymnema were used, which could contribute to variations in GA content. Another study by Suwan et al.^16^ mentioned that PCD leaves extract using ethanol as an extractant produced approximately 40,000 mg/kg dried weight of GA, which was about 5 times higher compared to the present study’s results with a %YGA of 4%. The higher yield in Suwan et al.’s^16^ study may be attributed to the prolonged extraction period of 3 days compared to the 2 h used in the current study. Furthermore, the authors point out that cultivated PCD from different regions may have varying GA content^16^, which could explain the relatively lower GA content observed in the current study. This indicates that environmental factors and cultivation practices might influence the GA content in PCD leaves. It was also observed that increasing the ethanol concentration from 50 to 60% (*v/v*) led to a significant drop in GA content. This finding aligns with the research conducted by Srinuanchai et al.^27^, who studied *G. inodorum* leaves and found that 75% (*v/v*) ethanol resulted in higher GA content compared to 95% (*v/v*) ethanol. The addition of water to ethanol was suggested as a factor that enhances the solubilizing capacity and improves the surface area for GA-ethanol interaction, facilitating the extraction process^73^. It is worth noting that glycoside molecules like GA are generally more readily extracted in aqueous solvents than alcoholic solvents^14^. Because, glycosides like gymnemic acid are polar compounds due to the presence of hydroxyl groups and other polar functional groups. Ethanol, on the other hand, is a polar solvent due to its ability to form hydrogen bonds with other polar molecules. In a 50% (*v/v*) ethanol solution, the presence of water also contributes to its polarity. When PCD leaves extracted with a 50% (*v/v*) ethanol solvent, hydrogen bonding and other intermolecular interactions can occur between the hydroxyl groups of GA and the hydroxyl groups of ethanol and water in the solvent. This interaction could have facilitated the dissolution of GA in the solvent. Based on the results, the study suggests that a concentration of 50% (*v/v*) ethanol provided the highest GA yield compared to distilled water and higher ethanol concentrations.

The study found that increasing the extraction temperature from 40 to 70 °C led to a significant increase in GA concentration. This observation is consistent with other research that has shown an increase in the recovery of phenolic compounds with the increase in extraction temperature, up to a certain point^74^. Higher extraction temperatures enhance solubility, diffusion rate, mass transfer rate, and surface tension, which improve the extractability of phenolic compounds^75^. These factors contribute to the increased extraction of GA from the PCD leaves at temperatures up to 70 °C. However, when the extraction temperature was set at 80 °C, there was a significant decrease in GA content compared to 70 °C. The decrease in GA content at 80 °C can be attributed to the degradation of heat-sensitive GA as the temperature increases. This phenomenon is supported by the findings of a study by Manika et al.^73^, where drying Gymnema leaves using sun drying drastically reduced GA content by 53% compared to shade drying. This suggests that high temperatures can lead to the degradation of GA. The authors also suggest that exposure to light, in addition to temperature, may have a deleterious effect on GA concentration^73^. This implies that protecting the extract from light during the extraction process is important to preserve the GA content. Similar observations have been made in studies on the extraction of phenolic compounds, where increasing extraction temperatures resulted in decreased total phenolic content^76,77^. This suggests that the degradation or loss of phenolic compounds can occur at higher temperatures.

The results indicate an inverse relationship between the amount of maltodextrin used and the GA content in the extract powder. These findings are consistent with other studies that have shown that an increase in maltodextrin concentration leads to a reduction in polyphenols, flavonoids, and antioxidant capacity in end products^78–80^. The decreasing trend in GA content with increasing maltodextrin concentration could be attributed to the dilution effect caused by maltodextrin, which reduces the concentration of GA in the extract^79^. The results indicate that increasing the concentration of maltodextrin leads to a decrease in GA content, likely due to dilution effects. The results also suggest that the inclusion of maltodextrin, even at a low concentration, can improve the physicochemical stability of the powder during storage. It's important to note that maintaining the physicochemical state of the dried product is crucial to preserve powder quality during storage^81^. In this context, using a relatively low concentration of maltodextrin, such as 0.5% (*w/v*), as an anti-caking agent during the drying process is recommended. This concentration not only retains the powder quality during storage but also improves solubility.

The characteristics of PCD extract powder prepared with maltodextrin was compared with previous research in terms of color parameters, moisture content, a_w_, GA content, and chlorophyll. Color analysis reveals that the PCD extract powder obtained in the current study had a lower brightness (L*) value compared to leaves dried at 50 °C, indicating a darker appearance of the final product. This darker appearance can be attributed to the addition of maltodextrin and the higher temperature (65 °C) used during the drying process. Previous research Binnisha et al.^61^, also reported similar findings, where Gymnema leaves powder dried at low temperatures resulted in increased brightness, greenness, and yellowness. The degree of greenness in dried leaves is influenced by the content of chlorophylls, which are susceptible to heat and light^82^. Chlorophyll a is more sensitive to degradation (pheophytization) than chlorophyll b. In the current study, the PCD extract powder contained relatively low amounts of chlorophyll a and chlorophyll b compared to the results reported by Binnisha et al.^61^. The usage of a solar dryer in their study minimized the destruction of chlorophylls during drying. On the other hand, the concentration of GA in the PCD extract powder was found to be higher in the current study compared to Binnisha et al.’s study^61^. This difference in GA content could be attributed to variations in the plant species used in the studies. Similarly, Jeytawan et al.^62^ reported the lowest concentrations of gymnemic acid in roasted *G. inodorum* leaves and suggested that variations in gymnemic acid content might be due to differences in the regions where the samples were obtained. The decrease in greenness observed in the PCD extract powder can be attributed to the conversion of chlorophylls into pheophytin and pyropheophytin during the drying process at high temperatures in the presence of maltodextrin^71^. However, it is worth noting that chlorophyll degradation products could still possess health effects similar to chlorophylls in terms of nutritional benefits^82^. Regarding moisture content and a_w_, the obtained data aligns with the results of other studies, which reported moisture content values lower than 10% (w/w) for extract powders^30,83^. A low a_w_ value (0.20–0.40) indicates stability against browning, lipid oxidation, microbial growth, hydrolysis, and enzymatic reactions^84^. It is a measure of available free water responsible for chemical, biochemical changes, and microbiological growth^84^. In the current study, the PCD powder with its low moisture content and a low a_w_ provides conditions that are sufficient to prevent the growth of various microorganisms and are considered ideal for dry or powdered products^85^.

In recent years, the DPPH scavenging activity assay has emerged as a widely employed method for evaluating antioxidant activity, despite the availability of other techniques such as ABTS, reducing power, and FRAP methods. Its popularity stems from several factors, including its simplicity, affordability, and rapidity. Moreover, the DPPH assay offers versatility by enabling the assessment of diverse sample types, encompassing natural extracts, synthetic compounds, and food products^86,87^. According to the present study, PCD extract powder exhibited a DPPH scavenging activity of 75.8%. This indicates its ability to neutralize DPPH free radicals. On the other hand, Subramanian et al.^23^, Fazal et al.^63^, Ghous et al.^64^ and Jeytawan et al.^62^ obtained radical scavenging activity of 73.4, 54.4, 83.0 and 62.6% respectively (Table 3). Jeytawan et al.^62^ stated that antioxidant capacity of processed leaves was higher when compared to fresh leaves. The authors suggested that lower antioxidant activity in fresh leaves might be due to the absence of dry and wet weight adjustment^62^. It is also interesting to note that the difference in scavenging activity between different studies might be due to variation in plant species, source of plant collection, type of solvent used for extraction and the extraction parameters.

## Conclusion

Although the use of herbal extracts to manage diabetes and other ailments has been long practiced, its utilization in its native form hinders its popularity due to low concentration of active substances, poor absorption, lack of standardization and other characteristics. Thus, preparing a formulation in a form that is acceptable with enriched gymnemic acid will open up interesting applications in the nutraceutical, functional food and therapeutical sectors. In this connection, the present study evaluated the conditions for the preparation of GA-enriched extracts from PCD leaves and their formulation into a powder form with maltodextrin as an anti-caking agent. The results also showed that the PCD extract powder had high radical scavenging activity which is very crucial to destroy the deleterious free radicals in diabetes. Yet, further investigation to assess other specific antidiabetic assays, combine with other herbs for synergistic effects, determine efficient extraction, and apply alternative drying/extraction techniques could be considered in the future. It can be anticipated that when the outcome of the study realized in the future in numerous applications, farmers could prefer the Gymnema cultivation as an alternative cropping choice against sugarcane, rice and corn as disposal of their residues through open burning causes fine PM 2.5 air pollution.

## Data Availability

The data sets generated during and/or analysed during the current study are available from the corresponding authors on reasonable request.

## Abbreviations

PCD: Phak Chiang Da
GA: Gymnemic acid
DPPH: 2,2-Diphenyl-1-picrylhydrazyl
a_w_: Water activity
*v/v*: Volume/volume
*w/v*: Weight/volume
AOAC: Association of Official Analytical Chemists
HPLC: High performance liquid chromatography
%Y_GA_: Mass yield percentage of extracted gymnemic acid
% P_Ext/Leaves_: Improved purity percentage of GA content in PCD extract powder relative to dried PCD leaves powder
%RSA: Percentage of radical scavenging activity
ANOVA: Analysis of variance
DMRT: Duncan’s multiple range test
